# Antipyretic, Antinociceptive, and Anti-Inflammatory Activities from *Pogostemon benghalensis* Leaf Extract in Experimental Wister Rats

**DOI:** 10.3390/medicines6040096

**Published:** 2019-09-20

**Authors:** Sushant Aryal, Balkrishna Adhikari, Kasmira Panthi, Pramod Aryal, Shyam Kumar Mallik, Ram Prasad Bhusal, Bahare Salehi, William N. Setzer, Javad Sharifi-Rad, Niranjan Koirala

**Affiliations:** 1 Department of Pharmacy, Universal College of Medical Sciences, Tribhuvan University, Bhairahawa, Rupandehi 32900, Nepal; sushantarl23@gmail.com (S.A.);; 2 Department of Natural Products Research, Dr. Koirala Research Institute for Biotechnology and Biodiversity, Kathmandu 44600, Nepal; 3 Department of Biochemistry and Molecular Biology, Monash University, Clayton, VIC 3800, Australia; 4 Department of Pharmacy, Purbanchal University College of Medical and Allied Sciences, Biratnagar, Morang 56613, Nepal; 5 Student Research Committee, School of Medicine, Bam University of Medical Sciences, Bam 44340847, Iran; 6 Department of Chemistry, University of Alabama in Huntsville, Huntsville, AL 35899, USA; 7 Aromatic Plant Research Center, 230 N 1200 E, Suite 100, Lehi, UT 84043, USA; 8 Zabol Medicinal Plants Research Center, Zabol University of Medical Sciences, Zabol 61615–585, Iran; 9 Department of Civil and Environmental Engineering, Faculty of Science and Technology, University of Macau, Macau SAR 999078, China

**Keywords:** *Pogostemon benghalensis*, antipyretic, antinociceptive, anti-inflammatory, PBME, methanolic extract

## Abstract

**Background:***Pogostemon benghalensis* leaves have traditionally been utilized for relieving body aches, headaches and fever. Based on its uses, the present study was designed to investigate the antinociceptive, antipyretic and anti-edematogenic activities from *P. benghalensis* leaves’ methanol extract (PBME) in Wister rats. **Methods:** The thermal (hot plate) and chemical (acetic acid-induced writhing and formalin test) models for antinociceptive effects, and the Brewer’s yeast induced hyperthermia test for antipyretic action and rat paw edema by carrageenan for anti-edematogenic activity, were applied for PBME at different dose levels. The acute toxicity of PBME through the oral route was performed to determine the lethal dose. **Results:** PBME significantly and dose-dependently reduced pyrexia and diminished edema volume, which depicted its antipyretic and anti-edematogenic effects respectively. The inhibition of writhing reflex, increased reaction latency and reduced frequency of licking indicated that PBME has significant dose-dependent antinociceptive activity. *P. benghalensis* methanol extract at 4000 mg/kg shows no sign of toxicity, which is a considerable, good margin of safety. **Conclusions:** The study illustrated the antipyretic, antinociceptive and anti-inflammatory potential of *P. benghalensis* leaf extract with a safety margin, and validated its traditional use to alleviate fever, pain, and inflammation.

## 1. Introduction

Pain, pyrexia, and inflammation are the primary indicators of many clinical conditions associated with injury, trauma, or infections [1]. Pain is defined by the International Association for the Study of Pain as, ‘an unpleasant sensory and emotional experience associated with actual or potential tissue damage, or described in terms of such damage’ [2]. Whereas, pyrexia is the characteristic defensive host mechanism of an increase in body temperature, occurring in response to pyrogenic infection, malignancy, or other diseases [3]. Inflammation is characterized by erythema, edema, heat, and pain initiated by the biological defensive mechanism to remove injurious stimuli [4]. Many therapeutic agents, such as NSAIDs, opioids, and steroids are available for the treatment of such disorders, but their potential side effects including bleeding, peptic ulcers, mental dependence, tolerance, and addiction, so have limited their clinical use [5]. Therefore, new plant-based drugs with low adverse effects are of current global research interest because plants are considered to be novel reservoirs [6].

Literature reports have widely recognized plant based herbal medicines for their better therapeutic value and less adverse effects compared to modern medicines [7,8]. Globally, around 80 per cent of the population in third world countries, including Nepal, relies on herbal remedies as a major form of health practice [9]. In addition, many developed nations from Europe, North America, and Australia have widely embraced the herbal-based complementary and alternative medicines (CAMs) as a part of their health care systems [10]. Nevertheless, the knowledge of traditional medicinal plants stills remains with indigenous people despite is global scope [11]. Investigation into the use of ethno-medicinal plants by scientists could be a valuable asset to discovering new medicinal products [12].

*Pogostemon bengalensis* from the Lamiaceae family is a small perennial shrub, 1–2 m tall, commonly known as Rudhilo in Nepal [13,14]. Traditionally, the leaf extract was used for the alleviation of fever [15] and its paste was given orally for the treatment of body pain [16]. The traditional formulation of leaves juices with sterculia gum and sesame oil are used in treatment of piles [17]. Moreover, pounded *P. bengalensis* leaves were inhaled and placed onto the forehead of patients for the treatments of coughs, colds, and headaches [18], suggesting the leaves to be a highly used part of the plant in folklore medicine. Phytochemical studies on P. bengalensis have shown the existence of phenols, flavonoids, alkaloids, saponins, terpenoids, and steroidal constituents as the principal active ingredients [19]. Scientifically, various medicinal properties have been reported, including antimicrobial activity against *E. coli*, *S. aureus*, *P. vulgaris*, and *A. parasiticus*; antifungal activity against *C. albicans* [20]; and antiviral against *S. virus* [21]. The extracts of *P. bengalensis* were investigated on ehrlich ascites carcinoma (EAC) induced mice, which significantly reduced the solid tumor volume [19].

Considering the use of *P. bengalensis* leaves in traditional folk remedies for pain, fever, and edema, this study is aimed to ascertain its pharmacotherapeutic value using experimental animal models.

## 2. Materials and Methods

### 2.1. Chemicals and Drugs

Carrageenan (S D Fine-Chem Limited, Mumbai, India), aspirin (Aristo Pharmaceuticals Pvt Ltd, Mumbai, India), indomethacin (Glenmark Pharmaceuticals, Mumbai, India), morphine (Modi Mundi Pharma Pvt. Ltd, New Delhi, India), Brewer’s yeast (Itanbiotech. Ltd, New Delhi, India), and naloxone (Samarth Life Sciences Pvt. Ltd., Mumbai, India) were purchased all from Indian companies. All reagents used in the research were of analytical grade.

### 2.2. Plant Material

The leaves of *Pogostemon benghalensis* (Burm.f.) Kuntze were collected on June 2016 from Gorkha, Nepal. The plant was authenticated from the National Botanical Garden, Department of Plant Resources, Ministry of Forest and Soil Conservation, Nepal. The voucher specimen (UHS1602) of collected plants was preserved in the Pharmacognosy Laboratory, Department of Pharmacy, Universal College of Medical Sciences, Tribhuvan University, Nepal.

### 2.3. Preparation of Plant Extract

The leaves collected from *P. bengalensis* were used for the preparation of methanol extract (PBME). Firstly, the raw leaves were dehydrated under shade and pulverized by mortar and pestle. The pulverized powder was macerated with methanol in an Erlenmeyer flask at room 25 °C for 15 days with intermittent agitation. The extract was filtered by a vacuum filter and concentrated in a rotavapor (Büchi Labortechnik, Essen, Germany) under reduced pressure and controlled temperature (40–50 °C) to give an extract with a 3.8% percentage yield, which was kept at 4 °C until further use.

### 2.4. Animals and Ethical Approval

Wistar rats of both sexes, weighing 150–250 g were obtained from the Animal House, Department of Pharmacy, Universal College of Medical Sciences, Tribhuvan University, Nepal. These animals had free access to standard feed and water ad libitum. They were kept in clean polypropylene cages with wooden dust (replaced every three days) under controlled temperature (22 ± 1 °C), maintaining a 12/12 h light/dark cycle. The animal care, handling and experimental protocols were carried out in strict compliance with official ethical guidelines of Nepal [8,22]. Animals were submitted to 35% CO_2_ euthanasia after completion of the study. All experimental procedures were approved by the Institutional Review Committee, Universal College of Medical Sciences, Tribhuvan University (Ref: UCMS/IRC/077/16). The approval date is 28 October 2016.

### 2.5. Acute Toxicity Studies

Organization of Economic Cooperation and Development (OECD) guidelines was adopted to carried out acute toxicity studies [23]. Animals were divided into five groups (*n* = 6): four test groups and a control group. The test groups were treated with PBME (500, 1000, 2000, and 4000 mg/kg, p.o.) at 10 mL/kg and a control with vehicle (saline). The any sign of toxic effects and mortality were observed every 1 h for the next 6 h and body weight was measured on day 1, 7 and 14 after treatments.

### 2.6. Acetic Acid-Induced Writhing Test

The test procedure followed the method as described by Pingsusaen et al., 2015 [24]. Six groups (*n* = 6) of rats were assigned for this experimental model; Group A: vehicle (control, saline, p.o.), Group B: indomethacin (10 mg/kg, p.o.), Groups C–E: PBME (100, 200, and 400 mg/kg, p.o., respectively), Group F: PBME (400 mg/kg, p.o.) + naloxone (5 mg/kg, p.o., treated 30 min prior of PBME administration). Thirty minutes post-dosing, 0.6% acetic acid (10 mL/kg) was injected intraperitoneally. The number of cumulative writhes was documented for 30 min. Inhibition percentage was calculated as in following equation: Inhibition (%) = ((A_Control_ – B_Test_)/ A_Control_) × 100, where, A is the mean writhes in control group and B is the mean writhes in test groups.

### 2.7. Thermal Test

The experiment followed the procedures reported by Sulaiman et al., 2008 [25]. Forty-two Wister rats were assigned to seven groups (*n* = 6). Group A: vehicle (control, saline, p.o.), Group B: morphine (5 mg/kg, p.o), Group C–E: PBME (100, 200, and 400 mg/kg, p.o.), Group F: PBME (400 mg/kg, p.o.) + naloxone (5 mg/kg, p.o.), and Group G: morphine (5 mg/kg, p.o) + naloxone (5 mg/kg, p.o.). Animals in Group F and G were treated with naloxone 30 min prior the administration of PBME or morphine. Thirty minutes post-dosing, each rat was placed in an Eddy’s hot-plate apparatus with a metal surface temperature of 55 ± 0.2 °C. Latency to discomfort (paw licking or jumping) was recorded by the time between placement in platform and the reaction provoked with 30 s cut-off time.

### 2.8. Formalin Test

The test procedure adopted the model as reported in the Sulaiman et al., 2008 literature [25]. Thirty-six rats were randomly assigned to six groups (*n* = 6). Group A: vehicle (control, saline, p.o.), Group B: morphine (5 mg/kg, p.o), Group C: aspirin (100 mg/kg, p.o.), Group D-F: PBME (100, 200, and 400 mg/kg, p.o.). Thirty minutes post-dosing, 2.5% (*v/v*) formalin (40% formaldehyde) in normal saline (50 μL/rat) was intraplantarly injected into the right paw, and the cumulative duration of licking was recorded at 5–10 min (neurogenic pain phase) and 20–30 min (inflammatory pain phase) post-formalin injection. The inhibition percentage was calculated as equation: Inhibition (%) = ((A_Control_ – B_Test_)/A_Control_) × 100, where, A and B are the duration of paw licking in the control group and test groups respectively.

### 2.9. Antipyretic Activity

Hyperthermia induced by Brewer’s yeast on Wister rats model was adopted to investigate the antipyretic action of *P. bengalensis* extract [24]. Initially, rectal temperatures of rats were recorded. Subcutaneously 20% w/v Brewer’s yeast suspension (10 mL/kg) was injected in each rats to induce hyperthermia. Eighteen hours post-injection, rectal temperature of each fasted-rats was recorded to ensure pyrexia. Accordingly, thirty rats were randomly assigned to five groups (*n* = 6). Group A: vehicle (control, saline, p.o.), Group B: paracetamol (100 mg/kg), and Group C–E: PBME (100, 200, and 400 mg/kg, p.o.). The rectal temperature was documented after 1, 2, 3, and 4 h post-dosing and antipyretic activity was expressed in term of °F.

### 2.10. Anti-Inflammatory Activity

To evaluate anti-inflammatory effect of *P. bengalensis* extract, a rat paw edema model induced by carrageenan was adopted [24]. Thirty selected Wister rats were randomly assigned to five groups (*n* = 6). Group A: vehicle (control, saline, p.o.), Group B: indomethacin (10 mg/kg p.o.), and Group C–E: PBME (100, 200, and 400 mg/kg, p.o.). Thirty minutes post-dosing, 50 µL of 1% (*w/v*) carrageenan was injected subplantarly into right hind paw. Change in paw volume was recorded for 6 h post- carrageenan injection and anti-edematogenic effect is expressed using the formula: final paw volume (1, 2, 3, 4 and 5 h) – initial paw volume (0 h).

### 2.11. Data Analysis 

The experimental outcomes are expressed as mean ± SD and statistical analysis was performed by GraphPad Prism 7 software. Multiple comparisons were performed with one-way ANOVA, followed by Dunnett’s test to determine the significance level in models, such as for nociceptive activity, Brewer’s yeast induced hyperthermia, and carrageenan-induced rat paw edema. *p*-values < 0.05 were considered significant.

## 3. Results

### 3.1. Acute Toxicity Study

At the maximum administered dose of PBME (4000 mg/kg), no mortality or toxicity symptoms was observed during the 14-day observation period. Therefore, oral administration of PBME had a good safety margin.

### 3.2. Acetic Acid-Induced Writhing Reflex

The PBME (100, 200, or 400 mg/kg, p.o.) showed a significant (* *p* < 0.05) and dose-dependent inhibition in the number of abdominal writhes (Table 1), with the percentage reduction ranging between 33% and 68%. The observed effect of PBME (400 mg/kg) was highly comparable to the effect of indomethacin (10 mg/kg), with no significant reverse action on the PBME (400 mg/kg) + naloxone (5 mg/kg) treated group.

### 3.3. Thermal Test

The PBME (100, 200, or 400 mg/kg, p.o.) also showed a significant (* *p* < 0.05) increase in pain threshold via thermally induced nociception model (Table 2). The dose-dependent rise in latency time was observed in PBME-dosing groups. The observed antinociceptive activity of PBME (400 mg/kg) was similar to that of morphine (5 mg/kg) with no significant reverse action on the PBME (400 mg/kg) + naloxone (5 mg/kg) treated group.

### 3.4. Formalin Test

Intra-plantar injection of 2.5% (*v/v*) formalin on the right paw increase the duration of licking (Table 3). However, the pre-dosing of rats with PBME (100, 200, or 400 mg/kg, p.o.) reduced the licking duration. The calculated percentage inhibition ranged from 49% to 73% in the neurogenic pain phase and 56% to 85% in the inflammatory pain phase. The comparisons revealed that the effect of PBME (400 mg/kg) was similar to that of morphine (5 mg/kg) on initial phase and aspirin (100 mg/kg) on late phase.

### 3.5. Antipyretic Effect

**Figure 1:**
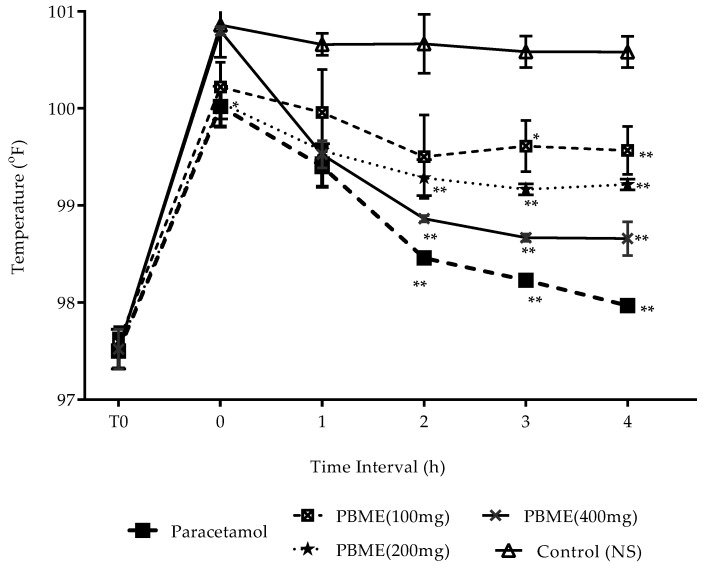
Antipyretic activity effect of *P. benghalensis* leaves’ methanol extract (PBME) and paracetamol in Brewer’s yeast induced hyperthermia rats. Values are mean ± SD (*n* = 6). *p*-value measured by ANOVA followed with Dunnett’s post hoc multiple comparisons. * *p* < 0.01, and ** *p* < 0.001 compared to control.

The PBME (100, 200, and 400 mg/kg p.o.) displayed a significant (* *p* < 0.05) decrease to elevated temperature in a dose-dependent manner (Figure 1). The antipyretic efficacy with the highest significant effect was observed at 2 and 3 h. The 400 mg/kg PBME demonstrated an antipyretic activity that was comparable to the positive control group paracetamol (100 mg/kg).

### 3.6. Anti-Inflammatory Activity

**Figure 2:**
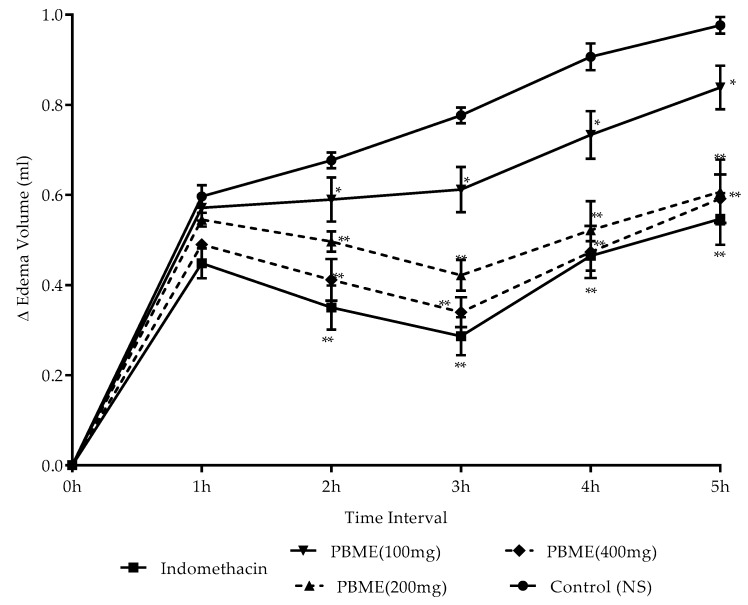
Difference (∆) edema volume (mL) on PBME, and indomethacin on rat paw edema induced by carrageenan. Values are mean ± SD (*n* = 6). *p*-value measured by ANOVA followed with Dunnett’s test post hoc multiple comparisons. * *p* < 0.01 and ** *p* < 0.001 compared to control.

Sub-plantar injection of 1% (*w/v*) carrageenan on the right hind paw increased the edema volume (Figure 2). However, the pre-dosing of rats with PBME (100, 200, or 400 mg/kg, p.o.) produced a significant (* *p* < 0.05) decrease in edema volume in a dose-dependent manner. The data revealed the efficacy of PBME (400 mg/kg) is slightly lower, but on a par to indomethacin (10 mg/kg), with highest reduction in paw volume being at 3 h post administration.

## 4. Discussion

With effective ethnopharmacological value, *P. bengalensis* is widely used by different tribes as an effective herbal medicine. Literature surveys have revealed that leaves of *P. bengalensis* are being used for alleviating headaches, fevers [15,18] and body pains [16]. This study observed the activity of *P. bengalensis* on pain, inflammation and pyrexia, to ascertain scientific rationales behind its ethnical practice. Our study validates the efficacy of methanol extract of *P. bengalensis* against induced pain, inflammation, and pyrexia. These useful biological activities may be attributed to tannins, flavonoids, alkaloids, glycosides, phenols, steroids, saponins, and terpenoids, as reported in previous phytochemical screenings [19].

The PBME has a wide safety margin. The lethal dose of PBME could not be acquired because no fetal reactions were detected at the highest dose, 4000 mg/kg. Three selective doses, 100, 200 and 400 mg/kg, were chosen for the in vivo study.

Fever, a major host defense mechanism is a brain-mediated response to respond on injury or infections with elevated body temperature [26]. This response is triggered by exo-pyrogens and facilitated by endo-pyrogens like TNF-α, IL-1β, IL-6, corticotrophin release factor (CRF), endothelin-1 (ET-1), preformed pyrogenic factor (PFPF), bradykinin, and prostaglandin E_2_ (PGE_2_) [27]. The ability of methanol extracted from *P. bengalensis* to reduce body temperature indicates the presence of components that can regulate either the PGE_2_-dependent fever activated cyclooxygenase (COX) in the preoptic zone of hypothalamus, or PGE_2_-independent fever activated by PFPF, CRF, and ET-1; or both [28]. The established literature reports suggests that alkaloids prohibit prostaglandin E_2_ [29] and flavonoids suppress TNF-α, [30]. Both of those compounds were reported in the leaf extracts of *P. bengalensis* [19]. Further investigation of those chemical compounds present in the plant leaf would be worthwhile, for determining the specific pathway of antipyretic activity.

Estimation of antinociceptive activity of plant based traditional medicine is a complex phenomenon. A single method is not precise enough to distinguish the origin and efficacy of the antinociceptive activity [31]. Therefore, a chemical and thermal nociception model was accessed during the experimental procedures. All the methods were used to distinguish between central or peripheral antinociceptive effect [32].

The acetic acid-induced nociception test is a non-specific pain model mediated by the prostaglandin pathway [33] to sensitize nociceptive neurons [34]. Pain stimulation from these processes are supposed to be responsible for abdominal contraction, forelimbs’ expansion and body elongation [35]. In this study, the extract’s antagonism of the acetic acid induced a writhing response probably resulted from either the blockage of the prostaglandin pathway or interference with the transduction cascade in efferent nociceptors [36]. Moreover, naloxone did not reverse the activity of PBME, suggesting that the effects do not depend entirely on opioid receptors. Phytochemicals in PBME may inactivate other endogenic pain stimulants responsible for nerve excitation that requires future investigation.

The thermal stimulus on rats is a highly sensitive method to evaluate central antinociceptive activity [37]. Paw licking and the jumping response on a warm surface are the components assessed here. Both are supraspinal reactions deactivated by central acting painkillers but not by peripheral anti-inflammatory drugs. In our study, *P. bengalensis* extract elevated reaction time, suggesting the presence of centrally active antinociceptive components. Since naloxone did not completely reverse the PBME action, opioid receptors did not completely facilitate the process, alhough it might be modulated by the components that act on ion channels and block the release of excitatory neurotransmitters involved in nociception [38].

The formalin-induced pain model characterized by early phase and late phase is a reliable method for possible mechanism studies [39]. The early neurogenic pain phase is stimulated by formalin to activate substance P after its injection. The late inflammatory pain phase is mediated through serotonin, histamine, prostaglandins, and bradykinin release [40]. The central antinociceptive agents equally suppress both stages, while peripheral agents inhibit only the later phase [34,41]. In our study, *P. bengalensis* extract reduced the duration of paw licking all phases, suggesting the presence of components that act like both central and peripheral antinociceptive drugs.

The observed antinociceptive activity of the extract in several test models anticipated the involvement of centrally and peripherally activating chemical constituents. However, the peripheral and central activities of the leaf extract were not reversed by naloxone, which confirmed the non-involvement of the opioid receptors for antinociceptive activity. It is therefore, reasonable to assume that the antinociceptive activity was mediated by inhibition of the COX pathway or modulation of ions channels involved in nociceptive neurons. This could be a highlight in the search for new analgesic components to substitute centrally acting opioids, which have unwanted side effects [42].

The rat paw swelling by carrageenan injection is a widely recognized biphasic model for anti-edematogenic study. The initial event (0–1 h) is local inflammation triggered by bradykinin, histamine, tachykinins, and reactive species. However, the late event (1–5 h) is linked with COX action, PGs, and neutrophil infiltration [24]. The *P. bengalensis* extract alleviated edema volume mostly in late phase (1–5 h), in a pattern similar to indomethacin. There are lines of evidence suggesting the anti-edema property of flavonoids, phenols, and terpenoids [43,44,45] which were found in PBME. *P. bengalensis* extract may inhibit the production of PGs or leukocytes in the anti-edematogenic process.

## 5. Conclusions

The assessment of the antipyretic, antinociceptive, and anti-inflammatory activities of methanol extract from *P. bengalensis* leaves shows that extracts have considerable effects. The study supports the traditional claims for fever, headache, and body pain management. The focus for the future is aimed towards isolation of chemical constituents from PBME. Results from the ongoing experiments will be reported in due course.

## Figures and Tables

**Table 1:** Result of *Pogostemon benghalensis* leaves’ extracts on the acid-induced writhing reflex.

Treatment Groups	Dose (mg/kg, p.o.)	No. Writhings	Inhibition %
Control		37.33 ± 1.03	
PBME	100	25.00 ± 1.41 *	33.03
	200	18.50 ± 1.05 *	50.44
	400	11.33 ± 0.81 *	69.65
PBME (400 mg) + naloxone (5 mg/kg)		13.11 ± 0.89	64.88
Indomethacin (10 mg/kg)	10	11.67± 1.21 *	68.74

*n* = 6; values are mean ± SD; *p*-value measured by ANOVA followed by Dunnett’s post hoc multiple comparisons. * *p* < 0.05 compared to the control group.

**Table 2:** Time course effect of *P. benghalensis* leaves’ extracts on the thermal model.

Sample	Dose (mg/kg, *p.o*)	Latency Time (min)				
		0	30	60	120	240
Control		14.8 ± 0.7	15.4 ± 1.0	15.2 ± 0.8	14.8 ± 1.0	15.2 ± 0.7
PBME	100	15.0 ± 1.1	16.1 ± 0.7	18.1 ± 1.1 *	18.4 ± 1.2 *^,#,¤^	17.3 ±1.2 *^,#,¤^
	200	15.2 ± 0.8	16.0 ± 1.41	17.6 ± 1.5 *	20.4 ± 1.2 *^,#,¤^	19.2 ± 1.5 *^#,¤^
	400	15.0 ± 0.5 *	21.0 ± 0.7 *	24.0 ± 0.7 *^,#^	26.0 ± 1.0 *^,#^	25.0 ± 0.9 *^,#^
Morphine	5	15.0 ± 1.2 *	22.0 ± 1.2 *^,#^	25.0 ± 1.6 *^,#,¤^	27.0 ± 0.8 *^,#,¤^	26.0 ± 1.1 *^,#^
PBME (400 mg) + naloxone (5mg/kg)		14.8 ± 0.7	20.4 ± 1.4 *^,#^	23.2 ± 0.7 *^,#^	24.9 ± 0.5 *^,#^	24.0 ± 0.5 *^,#^
Morphine (5 mg/kg) + naloxone (5 mg/kg)		15.0 ± 0.2 *^,¤^	15.3 ± 0.6 *^,¤^	15.2 ± 0.8 *^,¤^	15.1 ± 0.3 *^,¤^	15.1 ± 0.7 *^,¤^

*n* = 6. Values are mean ± SD; *p*-value measured by ANOVA followed by Dunnett’s post hoc multiple comparisons. * *p* < 0.05 compared to control. ^#^ *p* < 0.01 compared to morphine (5 mg/kg) + naloxone (5 mg/kg); ^¤^ *p* < 0.05 compared to PBME (400 mg) + naloxone (5 mg/kg).

**Table 3:** Result of *P. benghalensis* leaves’ extracts on the formalin test.

Sample	Dose (mg/kg, p.o.)	Duration of Licking (s) (Inhibition %)	
		Initial Phase (05–10 min)	Late Phase (20–30 min)
Control		66.0 ± 1.58	63.6 ± 3.28
PBME	100	34.0 ± 1.58 * (48.48)	27.8 ± 0.83 * (56.29)
	200	26.2± 0.83 * (60.303)	20.4 ± 1.51 * (67.925)
	400	17.4 ± 0.55 * (73.64)	9.40 ± 1.14 * (85.22)
Aspirin	100	63.0 ± 1.00 (04.54)	7.4 ± 1.14 * (88.36)
Morphine	5	15.8 ± 0.84 * (76.06)	9.8 ± 1.30 * (84.59)

*n* = 6, Values are mean ± SD. *p*-value measured by ANOVA followed with Dunnett’s post hoc multiple comparisons. * *p* < 0.05 compared to control.
